# Genes and nutrition, is personalised nutrition the next realistic step

**DOI:** 10.1186/2049-3258-72-S1-I1

**Published:** 2014-06-06

**Authors:** Christophe Matthys, Stefaan De Henauw, Patrick Kolsteren, Carl Lachat, John Van Camp, Kristin Verbeke, Nathalie Delzenne

**Affiliations:** 1 Clinical and Experimental Endocrinology, KU Leuven, B-3000 Leuven, Belgium; 2 Department of Public Health, Ghent University, B-9000 Ghent, Belgium; 3 Unit of Nutrition and Child Health, Institute for Tropical Medicine, B-2000 Antwerp, Belgium; 4 Department of food safety and food quality, Ghent University, B-9000 Ghent, Belgium; 5 Translational Research in GastroIntestinal Disorders, KU Leuven, B-3000 Leuven, Belgium; 6 Louvain Drug Research Institute, Metabolism and Nutrition Research Group, Université catholique de Louvain, B-1040 Brussels, Belgium

## 

Early 2014, the US Academy of Nutrition and Dietetics wrote in its position statement that “nutritional genomics provides insight into how diet and genotype interactions affect phenotype”[1] Nutrients can dictate phenotypic expression of an individual’s genotype by influencing the processes of gene transcription and protranscriptional processes (including translation). More important, the US Academy identified the practical application of nutritional genomics in the management of complex chronic disease as an emerging science [1]. Nutritional genomics is often presented as the new ‘holy grail’ in nutrition with an ultimate goal to establish a so-called personalised nutrition – i.e. an individual diet tailored to genotype-driven needs. However, one could wonder what the current state of the art is of this concept and to what extent it is realistic to expect such achievements in the near future.

During the fourth Belgian Nutrition Society Symposium the different aspects of the broad field of personalised nutrition have been discussed. New evidence of the importance of diet through the life-course is coming from epigenetics, i.e. changes in the regulation of the expression of gene activity without alteration of genetic structure [2]. There is now considerable evidence for nutritional epigenetic programming of biological functions. Impaired programming has been related to a wide range of phenotypes including obesity and diabetes. Prof Cnop covered in her talk the role of epigenetics in the development of obesity and diabetes.

A nutritionally relevant Single Nucleotide Polymorphism (SNP) is the C677T polymorphism. It is a common SNP of the methylenetetrahydrofolate reductase (MTHFR) gene, which encodes for the 5,10-MTHFR enzyme and uses folate to metabolize and thereby remove homocysteine. As homocysteine increase is considered a risk factor of cardiovascular diseases, the nutritional importance of this SNP is clear. Prof Helene McNulty has discussed in her talk the role of MTHFR, riboflavin and hypertension based on new and recently published data.

In December 2013, Science’s editors announced that research regarding the role of bacteria living inside the human body and their vital roles in determining how the body responds to challenges as malnutrition or cancer was one of the “Breakthroughs of the Year” [3]. In the same time period a letter in Nature was published which indicated the importance of the composition of the gut microbiota to explain the response towards nutritional intervention in obese individual [4,5]. In 2011, our annual meeting had been fully devoted to the relationship between the gut microbiota, nutrition and health (abstracts available at http://www.belgiannutritionsociety.be/data/userfiles/File/BNS2011-abstract-book.pdf). This year, Prof Jeroen Raes discussed in more depth the importance of the genome and the microbiome in human well-being.

Although the US Academy of Nutrition and Dietetics announced the practical application of nutritional genomics for complex chronic disease as an emerging science, it has recently been argued that personalised nutrition will not have the dramatic impact that was once expected [6]. Before personalised nutrition is part of the daily clinical practice further understanding of the complex influences of genetics and the interaction with diet is necessary. Nevertheless Prof Anne-Marie Minihane presented in detail the translational aspects of nutrigenomics. Finally Jo Goossens has discussed the possibilities of nutrigenomics from a business point of view.

This session has been assorted of oral presentations presented upon selection of abstracts, putting forward the dynamism of our young researchers in the field of nutrition and health in our country.
